# Short Sleep Duration on the Night Before Surgery Is Associated With Postoperative Cognitive Decline in Elderly Patients: A Prospective Cohort Study

**DOI:** 10.3389/fnagi.2021.821425

**Published:** 2022-01-28

**Authors:** Ayasa Takamino, Masakazu Kotoda, Yosuke Nakadate, Sohei Hishiyama, Tetsuya Iijima, Takashi Matsukawa

**Affiliations:** Department of Anesthesiology, Faculty of Medicine, University of Yamanashi, Chuo, Japan

**Keywords:** aging, cognitive dysfunction, elderly, postoperative complications, sleep

## Abstract

As the world is rapidly aging, and the number of elderly patients who undergo surgery is rising, postoperative cognitive decline among those patients has become an increasing healthcare problem. Although understanding the risk factors and mechanisms underlying the pathogenesis of postoperative cognitive decline is critically important from a preventative viewpoint, such knowledge and evidence are lacking. A growing body of evidence suggest an association between cognitive function and sleep duration. The purpose of this study was to investigate the association between postoperative cognitive function and sleep duration on the night before surgery using a wearable sleep tracker. In this 6-month prospective cohort study, we analyzed data from 194 patients aged ≥ 65 years who underwent elective non-cardiac and non-cranial surgery under general anesthesia. According to the sleep duration on the night before surgery, patients were categorized into following four groups: <5, 5–7, 7–9, and >9 h. Perioperative cognitive function and domains were assessed using a neuropsychological test battery, and the incidence and prevalence of cognitive decline over 6 months after surgery were analyzed using the multiple logistic regression analysis. During the 6-month follow-up period, 41 patients (21%) developed cognitive decline. The incidence of cognitive decline was significantly elevated for the patients with sleep duration < 5 h (vs. 7–9 h; surgical duration-adjusted odds ratio, 3.50; 95% confidence interval, 1.20–10.2; *P* < 0.05). The association between sleep duration and prevalence of cognitive decline was limited to the early postoperative period (at 1 week and 1 month). Among the cognitive domains assessed, attentional function was significantly impaired in patients with a sleep duration < 5 h [vs. 7–9 h at 1 week; 4/37 (10.8%) vs. 0/73 (0%); *P* < 0.05]. In conclusion, sleep duration < 5 h on the night before surgery was significantly associated with worse attentional function after surgery and higher incidence of cognitive decline. The present results indicate that sleep deprivation on the night before surgery may have a temporary but significantly negative influence on the patient's postoperative cognitive function and is a potential target for preventing cognitive decline.

## Introduction

Cognitive decline after surgery and anesthesia is an increasing healthcare problem, especially among the elderly populations (Moller et al., 1998; Steinmetz et al., 2009; Krenk et al., 2014; Evered and Silbert, 2018; Li et al., 2021; Migirov et al., 2021). Although there is no formal definition in ICD-11 or DSM-5, this complication generally refers to a late-onset, long-term, and objectively measurable cognitive impairment compared to the preoperative baseline, which is associated with poor patient outcomes, including prolonged hospital stay, reduced quality of life, and increased morbidity and mortality (Moller et al., 1998; Phillips-Bute et al., 2006; Steinmetz et al., 2009; Evered and Silbert, 2018).

In 2018, for the first time in human history, individuals over the age of 65 have outnumbered those aged < 5 years globally (United Nations, 2019). The rapidly aging population is a worldwide phenomenon, and the number of elderly people is projected to double by 2050, representing significant numbers of people at risk for cognitive decline after surgery and anesthesia (United Nations, 2019). Once it has developed, the cognitive decline can persist for weeks and months after onset, and there is no established treatment (Moller et al., 1998; Steinmetz et al., 2009; Evered and Silbert, 2018). Therefore, understanding the risk factors and mechanisms underlying the pathogenesis of postoperative cognitive decline is critically important from a preventative viewpoint. However, such knowledge and evidence are lacking.

A growing body of evidence suggests that an association exists between cognitive function and sleep (Gogenur et al., 2007; Walker, 2008; Ohara et al., 2018; Ni et al., 2019; Ma et al., 2020). Sleep is an essential biological function that is crucial for normal cognitive, immune, and metabolic function and overall health (Alhola and Polo-Kantola, 2007; Walker, 2008; Tucker et al., 2010; Xie et al., 2013; Davies et al., 2014; Dzierzewski et al., 2018; Ni et al., 2019). Adults are recommended to sleep for at least 7 h per night (Consensus Conference et al., 2015; Hirshkowitz et al., 2015). As experienced in daily life and as demonstrated in the literature, even a single night of sleep deprivation can induce adverse changes in cognitive performance (Jennings et al., 2003; Alhola and Polo-Kantola, 2007). Although polysomnography has been widely used in medical research as a gold standard for assessing sleep, the unnatural sleeping environment and multiple wires attached to the subject often interfere with sleep (Toussaint et al., 1995; Hutchison et al., 2008; Martin and Hakim, 2011). Recent development of wearable digital sleep trackers allows researchers to assess patient's sleep in more natural environment without disturbing the patient's sleep (Toussaint et al., 1995; Hutchison et al., 2008; Martin and Hakim, 2011; Haghayegh et al., 2019).

Several previous clinical studies have identified a relationship between sleep problems after surgery and impaired postoperative cognitive function (Kain and Caldwell-Andrews, 2003; Gogenur et al., 2007; Walker, 2008). However, research focusing on the impact of preoperative sleep on postoperative cognitive function is lacking. An animal study has demonstrated that preoperative sleep deprivation aggravated surgery-induced neuroinflammation and cognitive impairment in aged mice (Ni et al., 2019), suggesting that preoperative sleep also plays an important role in the development of postoperative cognitive decline.

This study aimed to investigate the possible association between sleep deprivation on the night before surgery and postoperative cognitive function.

## Materials and Methods

### Study Design and Participants

This was a 6-month prospective cohort study conducted at the University of Yamanashi Hospital, Japan between April 2019 and September 2020. The study protocol was approved by the institutional review board and was registered prospectively (H30687). Patients aged ≥ 65 years who underwent elective non-cardiac and non-cranial surgery under general anesthesia between April 2019 and March 2020, and without a history of dementia or previous general anesthesia within 6 months, were approached based on the availability of a trained study personnel during the patients' preoperative clinic visit. Written informed consent was obtained from the patients prior to their inclusion into the study, and they did not receive any financial compensation. This study adheres to the Strengthening the Reporting of Observational Studies in Epidemiology (STROBE) reporting guideline.

### Study Procedure

At the preoperative clinic visit, the Mini-Mental State Examination (MMSE) was conducted by the trained study personnel to evaluate preexisting cognitive impairment in all patients. A cognitive test battery [Rey auditory verbal learning test (Brand and Jolles, 1985), Trail-making test A (Delis et al., 2001), Letter fluency test (Benton, 1968), and Category fluency test (Lezak et al., 2012)] was also conducted in all patients to evaluate their baseline cognitive function (memory, attention, letter fluency, and category fluency, respectively). The following preoperative information and data were obtained from the patients or their medical records: age, sex, height, weight, BMI, presence of diabetes mellitus, hypertension, and depression, use of psychoactive medication (hypnotics, antipsychotics, antidepressants, anxiolytics, mood stabilizers) and/or antihistamine drugs, smoking habits, history of cerebrovascular accident, American Society of Anesthesiologists physical status (ASA-PS) classification, Pittsburg Sleep Quality Index, and serum C-reactive protein level. On the night before surgery, sleep duration was measured using a wearable sleep tracker (Fitbit Alta HR, Fitbit, San Francisco, CA), and the patients were categorized into the following four groups according to the sleep duration on the night before surgery: <5, 5–7, 7–9, and >9 h. The cut-off time was determined based on the sleep duration recommendations published by the National Sleep Foundation, American Academy of Sleep Medicine, and Sleep Research Society (Consensus Conference et al., 2015; Hirshkowitz et al., 2015). The study personnel was blinded to the grouping. Individuals with missing sleep duration data, preexisting cognitive impairment (MMSE score < 24) (Moller et al., 1998), and/or ASA-PS > 3 were excluded from the study. After the surgery, the following surgical and intraoperative information and data were obtained from the anesthetic charts: duration of surgery, type and dose of general anesthesia, heart rate, arterial blood pressure, bispectral index, and end-tidal CO_2_. Postoperative cognitive function was monitored using the same test battery at 1 week, 1 month, 3 months, and 6 months after surgery.

### Study Outcomes

The primary outcome was the incidence of postoperative cognitive decline. Patients were considered to develop cognitive decline if their postoperative cognitive test scores at each time point decreased by >1 standard deviation (SD) of all the included patients' baselines scores in at least two tests (Daiello et al., 2019). Secondary outcome measures included the prevalence of cognitive decline at each time point and a significant decrease (>1 SD) in the scores of each cognitive test domain.

### Statistical Analysis

For the analysis of patients' characteristics, intergroup comparisons were analyzed using one-way analysis of variance followed by the Dunnett test for continuous values, Kruskal-Wallis test for ordinal values, and chi-squared test for dichotomous values, respectively. The incidence and prevalence of cognitive decline in each group were analyzed using logistic regression, and the group with 7–9 h sleep duration served as the reference group. We evaluated 4 different models: the non-adjusted model; adjusted model 1, adjusted for surgery duration; adjusted model 2, adjusted for surgery, age, and sex; adjusted model 3, adjusted for covariates included in model 2 plus MMSE score and the history of cerebrovascular accident. Correlations between each of the variables were confirmed to be <0.3 using Pearson's correlation test or Spearman's rank correlation test as appropriate to avoid multicollinearity. The prevalence of a significant decrease in each test score at each time point was analyzed using the Fisher exact test. The data are shown as the mean (SD) or median (IQR) as appropriate; dichotomous variables are expressed as frequencies (%). All statistical analyses were conducted using R Version 4.0.4 (R Foundation for Statistical Computing, Vienna, Austria). A two-tailed value of *P* < 0.05 was considered statistically significant.

## Results

**Figure 1:**
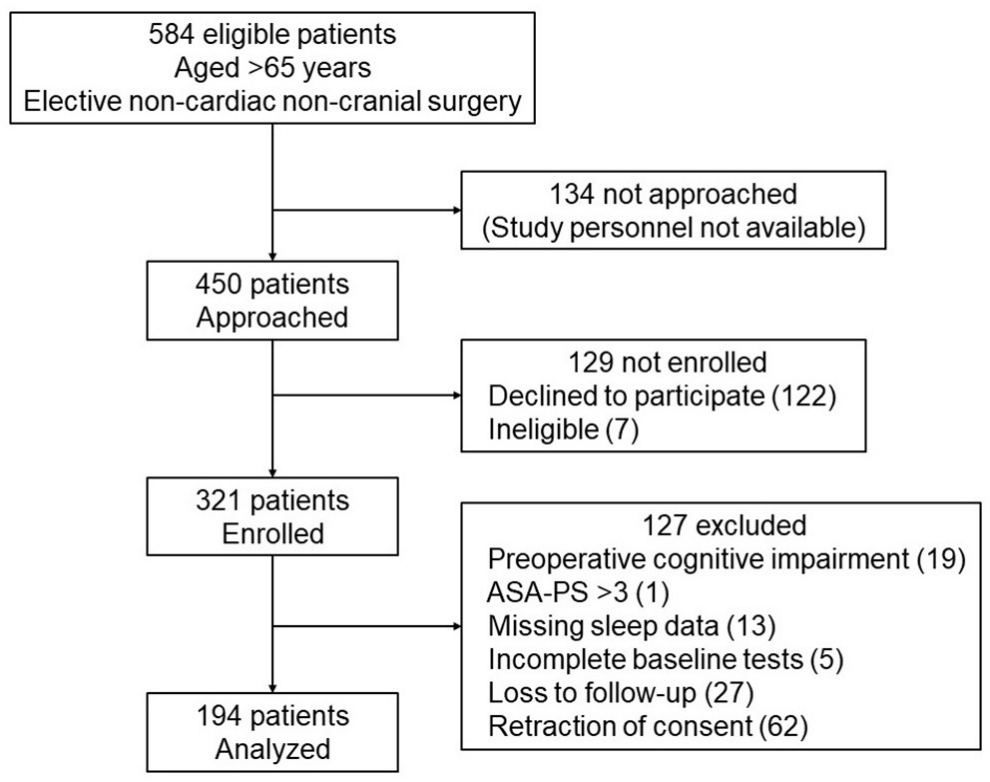
STROBE flow diagram for the included patients. Four hundred and fifty patients were approached for study participation, and 321 were enrolled after providing written informed consent. Subsequently, 127 patients were excluded, leaving 194 patients for the final analysis. ASA-PS, American Society of Anesthesiologists Physical Status.

Four hundred and fifty patients were approached for study participation, and 321 were enrolled after providing written informed consent. Subsequently, 127 patients were excluded because of the following reasons: preexisting cognitive impairment (*n* = 19), ASA-PS > 3 (*n* = 1), missing sleep duration data (*n* = 13), incomplete preoperative baseline cognitive tests (*n* = 5), loss to follow-up (*n* = 27), and retraction of consent (*n* = 62), leaving 194 patients for the final analysis (Figure 1). The mean age was 72.8 (5.3) years; 93 patients (48%) were women and 101 (52%) were men.

The descriptive statistics for the participants' perioperative characteristics according to sleep duration are summarized in Table 1.

**Table 1:** Participants' preoperative characteristics.

	**Sleep duration**					
**Variables**	**Total**	** <5 h**	**5–7 h**	**7–9 h**	**>9 h**	***P*** **-value**
	**(*n* = 194)**	**(*n* = 37)**	**(*n* = 66)**	**(*n* = 73)**	**(*n* = 18)**	
**General**						
Age, years	72.8 (5.3)	74.4 (6.3)	72.5 (4.8)	72.3 (4.7)	72.9 (6.4)	0.241
Female	93 (48%)	15 (41%)	32 (48%)	38 (52%)	8 (44%)	0.715
Height, cm	158.3 (9.0)	158.1 (8.8)	159.4 (1.0)	157.3 (8.0)	158.8 (10.4)	0.584
Weight, kg	59.0 (10.6)	58.3 (10.7)	59.8 (12.2)	58.1 (9.5)	61.0 (9.1)	0.647
BMI	23.5 (3.5)	23.3 (3.8)	23.4 (3.8)	23.5 (3.1)	24.3 (3.4)	0.798
ASA-PS	2 (2–3)	2 (2–3)	2 (2–3)	2 (2–2)	2 (2–2)	0.293
Cerebrovascular accident	12 (6.2%)	5 (13.5%)	3 (4.5%)	3 (4.1%)	1 (5.6%)	0.235
Diabetes	56 (28.9%)	13 (35.1%)	21 (31.8%)	18 (24.7%)	4 (22.2%)	0.576
Hypertension	88 (45.4%)	17 (45.9%)	29 (43.9%)	34 (46.6%)	8 (44.4%)	0.996
Smoking	84 (43.3%)	19 (51.4%)	30 (45%)	30 (41%)	5 (28%)	0.400
Serum CRP, mg/L	0.40 (1.11)	0.63 (1.28)	0.53 (1.59)	0.20 (0.25)	0.33 (0.44)	0.222
Hypnotics	23 (11.9%)	4 (10.8%)	9 (13.6%)	8 (11.0%)	2 (11.1%)	0.962
Antipsychotics	0 (0%)	0 (0%)	0 (0%)	0 (0%)	0 (0%)	NA
Antidepressants	2 (1.0%)	1 (2.7%)	1 (2.7%)	0 (0%)	0 (0%)	0.549
Anxiolytics	2 (1.0%)	1 (2.7%)	1 (2.7%)	0 (0%)	0 (0%)	0.549
Mood stabilizers	0 (0%)	0 (0%)	0 (0%)	0 (0%)	0 (0%)	NA
Antihistamine drug	7 (3.6%)	3 (8.1%)	2 (3.0%)	1 (1.4%)	1 (5.6%)	0.325
**Cognitive function**						
MMSE	27.9 (1.7)	28.0 (1.8)	28.0 (1.7)	28.0 (1.6)	27.3 (2.0)	0.429
Mild cognitive impairment (MMSE <28)	62 (32.0%)	14 (37.8%)	17 (25.8%)	24 (32.9%)	7 (38.9%)	0.525
RAVLT	40.3 (10.6)	38.5 (9.9)	40.5 (10.1)	41.9 (10.2)	36.9 (14.1)	0.206
TMT	48.9 (28.3)	59.2 (39.2)	49.5 (30.1)	44.6 (20.9)	43.0 (12.9)	0.058
LFT	9.6 (3.5)	8.6 (3.5)^a^	9.4 (3.4)	10.4 (3.6)	8.7 (3.0)	0.044
CFT	15.5 (4.2)	15.4 (4.1)	15.3 (4.6)	15.7 (4.1)	14.9 (3.6)	0.864
**Sleep**						
PSQ index	5.4 (3.4)	5.3 (3.4)	5.5 (3.8)	5.2 (3.3)	5.8 (2.6)	0.914
Daily sleep duration, min	386 (80)	384 (89)	381 (77)	397 (79)	365 (75)	0.423
Sleep duration on the night before surgery, min	398 (113)	223 (61)^b^	272 (32)^b^	464 (30)	589 (50)^b^	<0.001
**Surgery**						
Surgical duration, min	249 (154)	298 (200)^b^	207 (133)	155 (106)	105 (89)	<0.001
**Type of general anesthesia**						
Propofol	98 (50.5%)	15 (40.5%)	36 (54.5%)	39 (53.4%)	8 (44.4%)	0.491
Desflurane	73 (37.6%)	15 (40.5%)	25 (37.9%)	26 (35.6%)	7 (38.9%)	0.965
Sevoflurane	23 (11.9%)	7 (18.9%)	5 (7.6%)	8 (11.0%)	3 (16.7%)	0.337
**Dose**						
Propofol, mg		1,187 (690)	1,148 (632)	1,080 (500)	699 (234)^c^	0.019
Desflurane, mL		200 (94)^b^	135 (71)	110 (61)	93 (74)	<0.001
Sevoflurane, mL		95 (48)^b^	63 (27)^d^	47 (30)	29 (17)	<0.001
**Hemodynamic and physiological parameters**						
mHR, beats/min	62 (9)	62 (9)	61 (9)	61 (9)	62 (9)	0.738
mBP, mmHg	72 (11)	72 (14)	72 (8)	74 (12)	72 (5)	0.528
mBIS	46 (6)	46 (5)	45 (4)	47 (8)	47 (6)	0.280
mEtCO_2_, cmH_2_O	39 (3)	39 (3)	39 (3)	39 (3)	38 (2)	0.367

*ASA-PS, ASA Physical Status; CRP, C-reactive protein; MMSE, Mini Mental State Examination; RAVLT, Rey auditory verbal learning test; TMT, Trail making test; LFT, Letter fluency test; CFT, Category fluency test; PSQ, Pittsburg Sleep Quality; SD, standard deviation; mean HR, mean heart rate; mBP, mean blood pressure; mBIS, mean bispectral index; mEtCO_2_, mean end-tidal CO_2_*.

a *Dunnett test (vs. 7–9 h), P = 0.0305*.

b *Dunnett test (vs. 7–9 h), P <0.001*.

c *Dunnett test (vs. 7–9 h), P = 0.035*.

d *Dunnett test (vs. 7–9 h), P = 0.012*.

The preoperative general background and medical condition, including age, sex, height, weight, medical history, and ASA-PS, were not significantly different among the groups. Based on the MMSE results, there were no significant differences in the general baseline cognitive function and prevalence of mild cognitive impairment among the groups. Cognitive domains assessed using the test battery showed no significant differences between the groups except for the Letter fluency test scores, which were lower in the <5 h group than in the 7–9 h group [ <5 h: 8.6 (3.5) min, 7–9 h: 10.4 (3.6) min, *P* = 0.044]. Based on the Pittsburg Sleep Quality evaluation conducted during the patients' preoperative clinic visit, the baseline daily sleep quality including the self-reported daily sleep duration, was not significantly different between the groups. Regarding the surgical data, subjects with a sleep duration of <5 h on the night before surgery were significantly more likely to have a longer surgical duration and received larger doses of inhaled anesthetics than those with 7–9 h of sleep [surgical duration: <5 h: 298 (200) min, 7–9 h: 155 (106) min; dose of desflurane: <5 h: 200 (94) mL, 7–9 h: 110 (61) mL; dose of sevoflurane: <5 h: 95 (48) mL, 7–9 h: 47 (30) mL, all *P* < 0.001]. The mean heart rate, blood pressure, bispectral index, and end-tidal CO_2_ during surgery were not significantly different between the groups.

**Figure 2:**
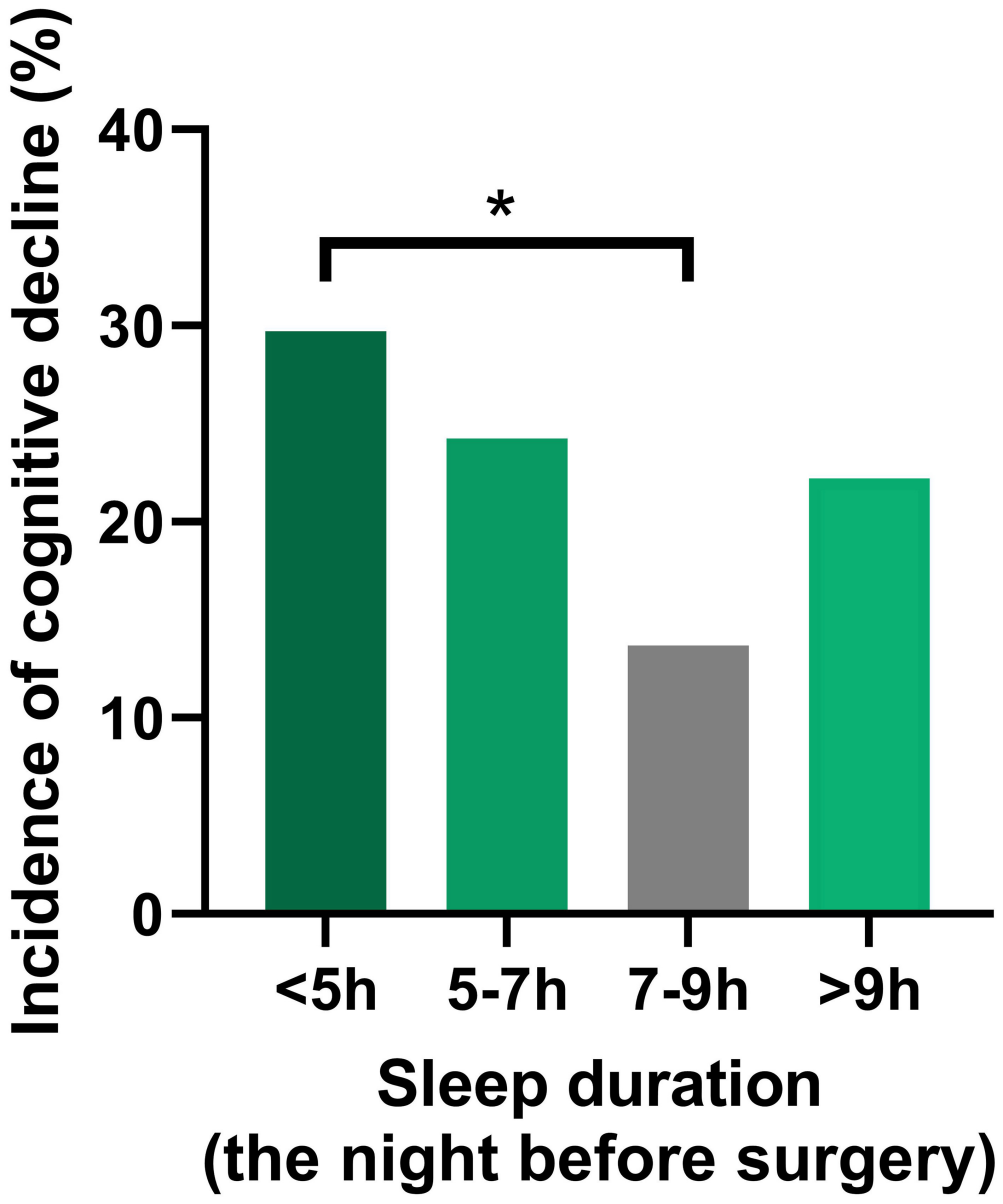
Association between sleep duration and the incidence of POCD. A U-shaped association was found between sleep duration on the night before surgery and incidence of POCD. The group with a sleep duration of 7–9 h exhibited the lowest incidence of POCD at 6 months after surgery. The logistic regression analysis demonstrated that the incidence of POCD was significantly elevated for the patients with a sleep duration <5 h, compared with the reference sleep duration (7–9 h). POCD, postoperative cognitive dysfunction. *P < 0.05.

During the 6-month follow-up period, 41 patients (21%) [male: 23 (22.8%), female: 18 (19.4%)] developed postoperative cognitive decline, but no patients died. Table 2 and Figure 2 show the results of the logistic regression analysis of the association between sleep duration and the incidence of cognitive decline according to daily sleep duration.

**Table 2:** Unadjusted and adjusted incidence of cognitive decline according to sleep duration.

	**Sleep duration**						
	** <5 h**		**5–7 h**		**7–9 h**	**>9 h**	
	**(*****n*** **=** **37)**		**(*****n*** **=** **66)**		**(*n* = 73)**	**(*****n*** **=** **18)**	
	**OR (95% CI)**	***P*** **-value**	**OR (95% CI)**	***P*** **-value**		**OR (95% CI)**	***P-*** **value**
**Incidence of postoperative cognitive decline**							
Unadjusted	2.67 (1.01–7.04)	0.048	2.02 (0.842–4.83)	0.115	Ref	1.8 (0.492–6.58)	0.374
Adjusted model 1 (surgical duration-adjusted)	3.50 (1.20– 10.2)	0.022	1.79 (0.730–4.38)	0.203	Ref	1.82 (0.484–6.86)	0.375
Adjusted model 2 (3 variables)	3.85 (1.26–11.8)	0.018	1.77 (0.723–4.35)	0.211	Ref	1.62 (0.404–6.48)	0.496
Adjusted model 3 (5 variables)	4.30 (1.20–12.5)	0.024	1.77 (0.715–4.40)	0.216	Ref	1.50 (0.360–6.29)	0.576

*Adjusted model 2: adjusted for surgical duration, age, and sex. Adjusted model 3: adjusted for covariates included in model 2 plus MMSE score and the history of cerebrovascular accident*.

The group with a sleep duration of 7–9 h exhibited the lowest incidence of cognitive decline at 6 months after surgery [10 of 73 (13.7%)]. The incidence of cognitive decline was significantly elevated for the patients with a very short sleep duration (<5 h) [surgery duration-adjusted odds ratio (95% CI): 2.67 (1.01–7.04), vs. 7–9 h, *P* < 0.05]. The significant association between a very short sleep duration and a high incidence of cognitive decline was unchanged in the unadjusted and adjusted logistic regression models (Table 2).

Table 3 shows the results of the multiple regression analysis of the association between sleep duration on the night before surgery and the prevalence of cognitive decline at each follow-up point. A very short sleep duration (<5 h) on the night before surgery significantly increased the prevalence of cognitive decline only at the early time points after surgery [surgery duration-adjusted odds ratio (95% CI): 11.8 (1.17–119) at 1 week; 15 (1.5–150) at 1 month, vs. 7–9 h, *P* < 0.05], but this association was not observed in the longer-term follow-up.

**Table 3:** Association between sleep duration and prevalence of cognitive decline at each time point.

	**Sleep duration**						
	** <5 h**		**5–7 h**		**7–9 h**	**>9 h**	
	**(*****n*** **=** **37)**		**(*****n*** **=** **66)**		**(*n* = 73)**	**(*****n*** **=** **18)**	
	**OR (95% CI)**	***P*** **-value**	**OR (95% CI)**	***P*** **-value**		**OR (95% CI)**	***P-*** **value**
**Prevalence of cognitive decline (surgical duration-adjusted)**							
1 week	11.8 (1.17–119)	0.036	2.78 (0.266–29.0)	0.393	Ref	4.32 (0.235–79.6)	0.325
1 month	15 (1.5–150)	0.021	3.58 (0.35–36.6)	0.282	Ref	6.1 (0.303–123)	0.238
3 months	1.89 (0.476–7.480)	0.366	1.98 (0.664–5.93)	0.220	Ref	1.99 (0.43–9.2)	0.379
6 months	2.71 (0.662–11.1)	0.166	1.68 (0.51–5.54)	0.394	Ref	0.949 (0.0993–9.08)	0.964

Table 4 shows the prevalence of a significant decrease (>1 SD) of the scores from the baseline in each cognitive test in the early postoperative period (at 1 week and 1 month).

**Table 4:** Prevalence of a significant decrease in each test score (>1 SD from the baseline).

	**Sleep duration**				
	** <5 h**	**5–7 h**	**7–9 h**	**>9 h**	
	**(*n* = 37)**	**(*n* = 66)**	**(*n* = 73)**	**(*n* = 18)**	
**Number of subjects who showed significant decrease in each test score**					
**1 week**					***P*** **-value**
RAVLT (memory)	1 (2.7%)	2 (3.0%)	0 (0%)	0 (0%)	0.422
TMT (attention)	4 (10.8%)	4 (6.1%)	0 (0%)	1 (5.6%)	0.023
LFT (letter fluency)	4 (10.8%)	3 (4.5%)	9 (12.3%)	2 (11.1%)	0.354
CFT (category fluency)	10 (27.0%)	11 (16.7%)	8 (11.0%)	3 (16.7%)	0.210
**1 month**					***P*** **-value**
RAVLT (memory)	0 (0%)	0 (0%)	0 (0%)	0 (0%)	NA
TMT (attention)	3 (8.1%)	6 (9.1%)	2 (2.7%)	1 (5.6%)	0.377
LFT (letter fluency)	2 (5.4%)	3 (4.5%)	8 (11.0%)	2 (11.1%)	0.422
CFT (category fluency)	12 (32%)	15 (23%)	9 (12%)	5 (28%)	0.065

*RAVLT, Rey auditory verbal learning test; TMT, Trail-making test; LFT, Letter fluency test; CFT, Category fluency test*.

Among the tests conducted, a decreased Trail making test score was significantly associated with the sleep duration [prevalence of a significant decrease of the score, 5 h: 4/37 (10.8%), 5–7 h: 4/66 (6.1%), 7–9 h: 0/73 (0%), >9 h: 0/18 (0%) at 1 week after surgery, *P* < 0.05].

## Discussion

Sleep plays critical roles in cognitive function and energy restoration, and it is well established that lack of sleep has a negative impact on the executive function and overall health of individuals (Jennings et al., 2003; Alhola and Polo-Kantola, 2007; Walker, 2008; Tucker et al., 2010; Xie et al., 2013; Davies et al., 2014). In the present study, we found a significant association between short sleep duration and worse perioperative cognitive function, compared with the reference sleep duration (7–9 h). In the postoperative period, the association between short preoperative sleep duration and higher incidence of postoperative cognitive decline was limited to the early postoperative period and was not observed during the long-term follow-up period. Although the association was temporary, our results may indicate that preoperative short sleep duration negatively influenced cognitive function after surgery and may have contributed to the development of cognitive decline.

Among the cognitive domains assessed, we found that attentional function was significantly impaired after surgery in patients with extremely short sleep durations (<5 h), which likely contributed to the high incidence of cognitive decline in those patients. Our findings are consistent with the results of earlier studies that have demonstrated that attentional function is the most likely cognitive domain to be impaired by acute sleep deprivation and can be affected by one night of sleep deprivation (Jennings et al., 2003; Alhola and Polo-Kantola, 2007).

Despite its importance and high impact on the patient's outcomes after surgery, the diagnostic criteria for postoperative cognitive decline are not yet established. A battery of several neuropsychological tests has been used to evaluate and define the patient's cognitive dysfunction. We used a battery of cognitive tests and conventional criteria for cognitive decline based on earlier studies (Benton, 1968; Brand and Jolles, 1985; Moller et al., 1998; Delis et al., 2001; Lezak et al., 2012). The overall incidence of cognitive decline at 6 months was 21%, which is consistent with previous studies that reported a 25.8% incidence at 1 week in elderly patients who underwent non-cardiac surgery (Moller et al., 1998).

There are various methods for assessing patients' sleep duration, including self-reporting, clinical interview, conventional polysomnography, and wearable sleep trackers. Although polysomnography has been widely used for objective sleep assessment, the unnatural sleeping environment and multiple wires attached to the subject often interfere with sleep (Toussaint et al., 1995; Hutchison et al., 2008; Martin and Hakim, 2011). Wearable sleep trackers are becoming increasingly popular in medical research settings as they have the advantage of assessing sleep duration in the natural sleep environment without disturbing the patient's sleep (Toussaint et al., 1995; Hutchison et al., 2008; Martin and Hakim, 2011; Haghayegh et al., 2019). Surgical patients are already nervous especially on the night before surgery. To avoid disturbing the patient's preoperative resting time and possible interference with their sleep, and to objectively assess the sleep duration independently from the patient's own assessment, we used up-to-date, unobtrusive wearable sleep trackers that have been shown to have high accuracy and consistency with polysomnography for measuring sleep duration (Haghayegh et al., 2019).

Although the precise mechanisms underlying the development of cognitive decline after surgery and anesthesia remain largely unknown, recent studies have indicated that neuroinflammation plays a pivotal role in the pathogenesis of cognitive decline (Androsova et al., 2015; Berger et al., 2019). Several clinical studies have reported elevated proinflammatory cytokine levels in the serum and cerebrospinal fluid of patients who developed cognitive impairment after surgery (Cape et al., 2014; Hirsch et al., 2016). Moreover, sleep plays crucial roles in the immune and nervous system, and sleep deprivation activates neuroinflammation in humans (Nadjar et al., 2017; Stokholm et al., 2017). Interestingly, in previous preclinical research using aged mice, sleep disturbance just before surgery enhanced surgery-induced neuroinflammation and consequently, cognitive dysfunction after surgery (Ni et al., 2019). In our study, short sleep duration on the night before surgery was significantly associated with a higher incidence of cognitive decline. It would be reasonable to assume that the sleep deprivation on the night before surgery accelerates surgery-induced cognitive impairment by enhancing neuroinflammation, and this influence persists until the neuroinflammation resolves.

In addition to sleep deprivation, previous studies have revealed that an excessively long sleep duration is associated with increased risks for cognitive decline and various diseases as well as death in the elderly population (Cappuccio et al., 2010; Shen et al., 2016; Yin et al., 2017; Jike et al., 2018; Ohara et al., 2018; Ma et al., 2020; Hua et al., 2021); a U-shaped association between sleep duration and cognitive function is a common finding among recent studies. Based on those findings, the National Sleep Foundation has announced that deviation from an ideal sleep duration— <5 h or more than 9 h for elderly people—is not recommended for individuals who are 65 years and older (Hirshkowitz et al., 2015). Consistently with those previous reports, we observed the lowest incidence of cognitive decline in patients with a sleep duration of 7–9 h and a U-shaped association between sleep duration on the night before surgery and the incidence of cognitive decline. Although the baseline scores in the MMSE and most of the cognitive tests were not significantly different between the groups, a similar U-shaped association was observed in most of the cognitive tests, indicating the similar association between preoperative cognitive function and sleep duration.

To the best of our knowledge, this is the first study to report an association between short sleep duration on the night before surgery and worse perioperative cognitive function and increased incidence of cognitive decline. Our findings suggest that preoperative sleep plays a critical role in the development of cognitive decline and is a potential target for preventing this complication.

This study has several limitations. First, we objectively assessed the sleep duration only on the night before surgery and not throughout the perioperative period. Therefore, the results could have contained confounding factors, such as patients' postoperative sleep duration. In addition, due to the observational study design, it could only demonstrate the association between the aforementioned conditions, and not the direct impact of short sleep on the incidence of postoperative cognitive decline. Furthermore, the present results also cannot demonstrate the impact of the short preoperative sleep or transient postoperative cognitive decline on other surgical outcomes, such as length of hospital stay or quality of life. Future studies should investigate the influence of preoperative short sleep and consequent transient cognitive decline on other surgical outcomes. Second, although the baseline MMSE scores were similar among the groups, there was a slight difference in baseline cognition according to sleep category. Therefore, there is the possibility that baseline neurodegenerative disease may be the etiology of the sleep alterations identified. We used the commonly used cutoff (-1SD) to detect significant decline in cognitive function. However, this approach resulted in loss of detailed information and may have influenced the results. In addition, depression and postoperative delirium—acute and short-term impairment of consciousness up to 7 days following surgery—were not the focus of the present study, and we did not evaluate patients for these conditions. However, the presence of depression and/or delirium during perioperative period could have affected the patients' sleep and/or neurocognitive performance. Third, although accelerometry approaches for evaluating sleep duration have shown to be accurate, indirectly-estimated sleep duration might not reflect the patient's actual sleep (Cook et al., 2019). We did not investigate the characteristics and pattern of the patients' sleep and the mechanism by which it changes or remains similar before and after surgery. Our future studies will focus on this aspect. In addition, compared with the reference group (sleep duration of 7–9 h), the group with the shortest sleep duration had longer surgical duration and received larger doses of anesthetic drugs. Although we adjusted the data for the surgical duration, the dose of anesthetics administered during surgery could have affected the patients' postoperative cognitive function. Finally, because many patients did not wish to participate or withdrew their consent before they completed the study, there might have been something different about the patients who declined to participate from those who completed. Further studies are needed to support and confirm the findings of the current study.

## Conclusions

Sleep duration < 5 h was significantly associated with worse perioperative cognitive function and a higher incidence of postoperative cognitive decline. The association between short sleep duration and the prevalence of cognitive decline was observed in the early postoperative period but not in the late period. The results indicate that sleep deprivation on the night before surgery may have a temporary but significant association with the patient's perioperative cognitive function and is a potential target for preventing cognitive decline after surgery.

## Data Availability Statement

The raw data supporting the conclusions of this article will be made available by the authors, without undue reservation.

## Ethics Statement

The studies involving human participants were reviewed and approved by University of Yamanashi, Faculty of Medicine, Research Ethics Board. The patients/participants provided their written informed consent to participate in this study.

## Author Contributions

AT designed the study, acquired, analyzed, and interpreted the data, and helped draft the manuscript. MK designed the study, analyzed, and interpreted the data, and drafted the manuscript. YN analyzed the data, interpreted the results, and revised the manuscript critically for important intellectual content. SH helped design the study and acquire the data and revised the manuscript critically for important intellectual content. TI helped design the study and collect the data and revised the manuscript critically for important intellectual content. TM supervised the study and interpreted the data and revised the manuscript critically for important intellectual content. All authors have provided final approval for this version of the report to be published.

## Conflict of Interest

The authors declare that the research was conducted in the absence of any commercial or financial relationships that could be construed as a potential conflict of interest.

## Publisher's Note

All claims expressed in this article are solely those of the authors and do not necessarily represent those of their affiliated organizations, or those of the publisher, the editors and the reviewers. Any product that may be evaluated in this article, or claim that may be made by its manufacturer, is not guaranteed or endorsed by the publisher.
